# Recruiting Unicellular Algae for the Mass Production of Nanostructured Perovskites

**DOI:** 10.1002/advs.202300355

**Published:** 2023-02-12

**Authors:** Lucas Kuhrts, Lukas Helmbrecht, Willem L. Noorduin, Darius Pohl, Xiaoxiao Sun, Alexander Palatnik, Cornelia Wetzker, Anne Jantschke, Michael Schlierf, Igor Zlotnikov

**Affiliations:** ^1^ B CUBE – Center for Molecular Bioengineering Dresden University of Technology Tatzberg 41 01307 Dresden Germany; ^2^ AMOLF Science Park 104 Amsterdam 1098 XG The Netherlands; ^3^ Van ‘t Hoff Institute for Molecular Sciences University of Amsterdam Amsterdam 1090 GD The Netherlands; ^4^ Dresden Center for Nanoanalysis (DCN) Center for Advancing Electronics Dresden (cfaed) Dresden University of Technology Helmholtzstraße 18 01069 Dresden Germany; ^5^ Helmholtz‐Zentrum Dresden Rossendorf Bautzner Landstraße 400 01328 Dresden Germany; ^6^ Dresden Integrated Center for Applied Physics and Photonic Materials Dresden University of Technology Nöthnitzer Str. 61 01187 Dresden Germany; ^7^ Light microscopy facility of the Center for Molecular and Cellular Bioengineering (CMCB) Dresden University of Technology 01062 Dresden Germany; ^8^ Institute for Geosciences Johannes Gutenberg University Mainz 55099 Mainz Germany; ^9^ Physics of Life DFG Cluster of Excellence TU Dresden 01062 Dresden Germany

**Keywords:** biological materials, perovskites, unicellular algae

## Abstract

Functional capacities of lead halide perovskites are strongly dependent on their morphology, crystallographic texture, and internal ultrastructure on the nano‐ and the meso‐scale. In the last decade, significant efforts are directed towards the development of novel synthesis routes that would overcome the morphological constraints provided by the physical and crystallographic properties of these materials. In contrast, various living organisms, such as unicellular algae, have the ability to mold biogenic crystals into a vast variety of intricate nano‐architectured shapes while keeping their single crystalline nature. Here, using the cell wall of the dinoflagellate *L. granifera* as a model, sustainably harvested biogenic calcite is successfully transformed into nano‐structured perovskites. Three variants of lead halide perovskites CH_3_NH_3_PbX_3_ are generated with X = Cl^−^, Br^−^ and I^−^; exhibiting emission peak‐wavelength ranging from blue, to green, to near‐infrared, respectively. The approach can be used for the mass production of nano‐architectured perovskites with desired morphological, textural and, consequently, physical properties exploiting the numerous templates provided by calcite forming unicellular organisms.

## Graphical abstract

Biologically formed calcium carbonate nanostructures are transformed into functional lead halide perovskite with controllable electro‐optical properties. This approach can be applied for the mass production of nano‐architectured perovskites with tunable physical characteristics that are inherited from the vast pool of structural and textural properties provided by unicellular calcitic algae, such as coccolithophores and dinoflagellates.

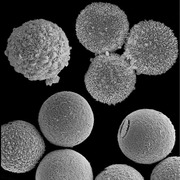

## 1 Introduction

The last decade of functional materials research has been immersed in the exploration of the vast possibilities offered by lead halide perovskites. The focus on organic‐inorganic perovskite compounds, having a solar‐cell power conversion rate exceeding 25%, stems from the relative simplicity and cost‐effectiveness of their production.^[^ ^1^, ^2^, ^3^, ^4^, ^5^, ^6^ ^]^ Together with their light‐emitting and electro‐optical properties, such as high photo‐luminescence quantum yield, lasing capacity and spectral tunability make perovskite materials highly attractive for numerous applications across scientific and technological domains.^[^ ^7^, ^8^, ^9^, ^10^, ^11^ ^]^

Tuning the properties of perovskites for various applications is usually achieved through compositional and structural control during the preparation of lead halide perovskites.^[^ ^12^, ^13^, ^14^, ^15^ ^]^ The strong structure‐property‐function relationship has been driving tremendous synthetic efforts to produce a variety of nano‐scale morphologies, such as rods, cubes, spheres, and 2D materials using different wet‐chemical and chemical vapor deposition approaches.^[^ ^10^, ^16^, ^17^, ^18^, ^19^ ^]^ Bottom‐up processes, while well‐controlled, are restricted to yield thermodynamically favorable cuboidal shapes, reflecting the crystal habit of lead halide perovskites having a cubic crystal structure.^[^ ^20^, ^21^ ^]^ As the result, although nano‐architectured structures are expected to provide enhanced performance,^[^ ^7^ ^]^ currently, we are still limited in our ability to produce morphologically and texturally complex perovskite assemblies.

In contrast, minerals formed by living organisms exhibit shapes that are considerably different from abiotically formed minerals. The characteristics of biologically produced mineral units comprising the various mineralized tissues, on all scales, from crystal lattice properties on the atomic scale to the shape of the entire unit on the macroscopic scale, reflect hundreds of millions of years of evolution under functional, ecological, and phylogenetic constraints.^[^ ^22^ ^]^ Here, the process of biomineralization is governed by the cellular components that orchestrate biomineral nucleation, growth and morphogenesis.

Biogenic calcium carbonate (CaCO_3_), calcite or aragonite—two of the most abundant biominerals in nature—is molded by various organisms into the most intricate nano‐architectured shapes while keeping its single crystalline nature.^[^ ^23^ ^]^ This is achieved by controlling the thermodynamics and kinetics of crystal growth through the introduction of transient phases, such as amorphous calcium carbonate,^[^ ^24^, ^25^ ^]^ and by the strict control of the physical and biochemical boundary conditions, such as geometric confinement, precursor concentrations and macromolecular or inorganic additives.^[^ ^26^ ^]^

Influenced by functional biological materials, the fields of biomineralization, bioprospecting, biomimicry and bioinspiration have become major scientific disciplines. Here, the scientific community aims to understand the principles of biomineral formation and function with the goal to implement this knowledge in the production of technologically relevant materials.^[^ ^27^, ^28^, ^29^, ^30^, ^31^ ^]^ However, aside from a few unique cases, such as conversion of silica in diatoms into other microporous structures,^[^ ^32^, ^33^ ^]^ most of these efforts use the natural products as a model system for the fundamental study of biomineralization or as organic templates for in vitro mineral microfabrication,^[^ ^34^, ^35^ ^]^ while the actual biological material remains unexploited.

Human history is saturated with examples of unsustainable use of biologically formed materials for numerous applications, from medicinal uses, jewelry, and fabrics to decorative and construction materials.^[^ ^36^, ^37^, ^38^ ^]^ In most cases, these practices led to significant depletion of natural resources, ecological damage and decline in biological diversity. This also applies to mineralizing animals, such as mollusks, that were extensively harvested for their strong shells and byssus fibers.^[^ ^39^ ^]^ However, in some cases, we also learned to harvest living organisms in a way that does not harm our environment at neutral pH and by fixing CO_2_. For example, in a scaled‐up photo‐bioreactor, microalgae production can reach up to 3.8 t of biomass per year.^[^ ^40^ ^]^ This includes unicellular algae, such as diatoms, coccolithophores and dinoflagellates that can be sustainably cultivated in considerable amounts.^[^ ^41^ ^]^

**Figure 1:**
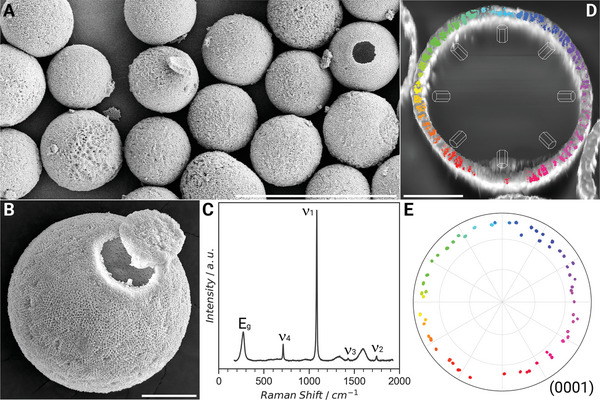
Calcitic shells of the dinoflagellate *L. granifera*. A) The calcitic shells of *L. granifera* exhibiting a porous ultrastructure. B) A single shell of *L. granifera*. C) Raman spectrum from a single shell confirming its calcitic nature. D) Electron back‐scattering diffraction (EBSD) map and E) a corresponding (0001) pole figure of a single polished shell demonstrating that it consists of single crystalline domains where the *c*‐axis of calcite is pointing radially from the center of the shell outwards. The colors in (E) correlate with similarly colored areas in (D). Scale bars: (A) 50 µm, (B) 5 µm and (D) is 5 µm.

These common phytoplankton types produce a staggering variety of highly complex mineralized cell walls with exquisite morphological control. Whereas in the case of diatoms, the mineral cell walls are made of amorphous silica^[^ ^42^ ^]^ and in coccolithophores, the walls consist of a number of single‐crystalline units made of calcite,^[^ ^43^ ^]^ the cell walls of some dinoflagellates, such as *Leonella granifera* (**Figure** **1**A,B), are made of a continuous calcitic spherical shell (Figure 1C).^[^ ^44^ ^]^ In this work, we adapt a recently developed ion exchange/insertion reaction^[^ ^45^ ^]^ and use the cell walls of *L. granifera* as a template for the production of functional lead halide perovskites with synthetically tunable nanostructured morphologies that inherit their crystallographic texture from the underlying calcitic shell.

## 2 Results and Discussion

Dinoflagellates have a number of different life stages. During the resting stage (cyst stage), they are able to survive up to 100 years in sediments until germination.^[^ ^46^, ^47^ ^]^ Whereas most dinoflagellate families produce organic‐walled cysts, in the Thoracosphaeraceae family, mineralized calcitic cell walls are formed. One example is the coccoid cells of *L. granifera* (Figure 1A,B) carrying a continuous spherical shell that is perforated by pores forming a highly ordered hexagonally packed ultrastructure.^[^ ^44^ ^]^ Here the mineralized cell walls are in the range of 10 to 15 µm in diameter, ≈2 µm in thickness and the diameter of the pores is in the range of 200 nm. Furthermore, the entire wall is divided into coherent crystallographic domains in which the *c*‐axis of calcite is pointing radially from the center of the cyst outwards (Figure 1D,E).

Solid‐state transformation of calcite into methylammonium halide perovskites (MAPbX_3_ with X = Cl^−^, Br^−^, I^−^, MA = CH_3_NH_3_^+^) requires a total exchange of all anions and cations in the original material. In a previously suggested synthetic route,^[^ ^45^ ^]^ this conversion was realized via two separate chemical exchange steps: first, from a carbonate salt (MCO_3_) to lead carbonate (PbCO_3_), and second, from lead carbonate to MAPbX_3_. In this study, dinoflagellates were prepared for chemical transformation by an initial cleaning step to remove all the organic content in the calcitic shells by bleaching and heat treatment at 600 °C. The replacement of Ca^2+^ was performed in excess of Pb^2+^ using a 24 m lead perchlorate (PbCl_2_O_8_) solution with the addition of either 10 vol% or 60 vol% of 1 m NaOH resulting in pH values of 1.3 and 2.4, respectively. This resulted in the formation of two intermediate materials: cerussite (PbCO_3_) and hydrocerussite (Pb_3_(CO_3_)_2_(OH)_2_), the basic form of lead carbonate, respectively.^[^ ^48^ ^]^ In order to overcome the low surface energy of calcite and ensure complete wetting of the nanostructured surface, the reactions were performed during centrifugation until maximum conversion.

**Figure 2:**
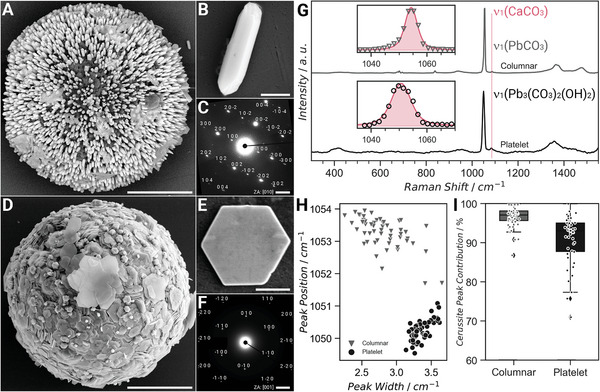
The first conversion step – the formation of lead carbonates. A,D) The morphology of the shells after the first conversation step leading to the formation of B) column‐ and E) platelet‐covered cerussite and hydrocerussite spheres, respectively. In both cases, the *c*‐axis of the corresponding C) orthorhombic cerussite crystal system (Zone Axis is [010]) and F) hexagonal hydrocerussite crystal system (Zone Axis is [001]) is pointing toward the center of the shell. G) Raman spectra of the converted algae confirming the transformation from calcite (1098 cm⁻^1^) to the corresponding H) lead carbonates (1050–1053 cm⁻^1^) exhibiting I) more than 90% cerussite peak contribution from integrating the respective Raman peak (inset in (G)) in both columnar and platelet structures. Scale bars: (A) 5 µm, (B) 500 nm, (C) 2 nm⁻^1^, (D) 5 µm, (E) 1 µm, and (F) 2 nm⁻^1^.

Scanning electron microscopy (SEM) images reveal the formation of two distinct morphologies formed after the first conversion step (**Figure** **2** and Figure S1, Supporting Information). Slender cerussite column‐like crystals (Figure 2A) emerged at a lower NaOH concentration with the [001] direction of their orthorhombic crystal system pointing toward the center of the spherical shells (Figure 2B,C). In contrast, hydrocerussite is formed at a higher NaOH concentration as hexagonal platelet‐like crystals (Figure 2D) with the [001] direction of the corresponding hexagonal crystal system oriented toward the center of the spheres (Figure 2E,F). These morphologies correspond to the expected crystal habit of both minerals, while their crystallographic orientation is most probably achieved through epitaxy on the *a‐b* plane of all three structures.^[^ ^48^, ^49^ ^]^ Here, the *a* lattice parameter of calcite is 4.99 nm, while that of cerussite and hydrocerussite is 5.18 and 5.24 nm, respectively. The relative lead carbonate yield was determined from the ratio of the integrated spatially resolved Raman signals from the internal CO_3_ vibration of calcite (1087 cm⁻^1^) and the two lead carbonates (1050 cm⁻^1^ for cerussite and 1053 cm⁻^1^ for hydrocerussite) (Figure 2G). Similar approach was used to determine the compositional contribution of all composite structures in this work. The formation of the two phases was confirmed by comparing the peak position and the peak width of the two differently ornamented spheres (Figure 2H). The presence of the specific lattice vibration peak for hydrocerussite at 400 cm⁻^1^ further confirms the predominance of this lead carbonate phase in platelet‐covered shells. For both morphologies, lead carbonate contribution of above 90% was realized (Figure 2I).

The synthesis approach used here is based on a study where the carbonate salt converted to lead halide perovskite was BaCO_3_.^[^ ^45^ ^]^ Whereas during the conversion of orthorhombic BaCO_3_ to orthorhombic cerussite, retention of initial shape was indicative of a cation exchange mechanism, the obvious overgrowth of lead carbonate onto trigonal calcite suggests a different reaction mechanism. In accordance with previous reports on the low concentration lead uptake by calcite,^[^ ^50^, ^51^, ^52^ ^]^ a solvent‐mediated transformation via an interface coupled dissolution reprecipitation (ICDR) offers a comprehensive mechanism explaining the obtained lead carbonate morphologies.

The ICDR mechanism describes the precipitation of a new phase within an interfacial fluid layer with physico‐chemical properties different from that of the bulk solution. When the biogenic calcite is in contact with the acidic lead solution (pH<2.4), ICDR is initiated by the dissolution of calcite into Ca^2+^ and CO_3_^2−^—a weak base which reacts with protons of the bulk solution. The pK_a_ of the equilibrium is ≈6.7,^[^ ^53^ ^]^ thus the pH of the interfacial fluid layer is significantly higher. Based on the increased pH level and the solubility products of calcite and lead carbonates, we can formulate the thermodynamic driving force and the kinetics of lead carbonate formation.

While at initial pH below 2.5 both phases are soluble, at a pH of up to 6.7 at the interface layer lead carbonate (*K_s_* (PbCO_3_)≈10⁻^14^ mol L⁻^1^) precipitates more readily compared to calcite (*K_s_* (CaCO_3_)≈10⁻^8^ mol L⁻^1^).^[^ ^54^ ^]^ Consequently, the extremely high concentration of lead ions influences the obtained morphologies in two ways. First, due to the very fast kinetics of lead carbonate precipitation at the given pH and concentration, the diffusion of CO_3_^2−^ ions from the boundary layer to the bulk solution is negligible and the transformation of calcite to lead carbonate remains coupled to the interfacial layer. Second, the high concentration of lead ions favors multiple nucleation events of new lead carbonate crystals, leading to the formation of small crystals of lead carbonate that overgrow the dissolving calcitic shell.

**Figure 3:**
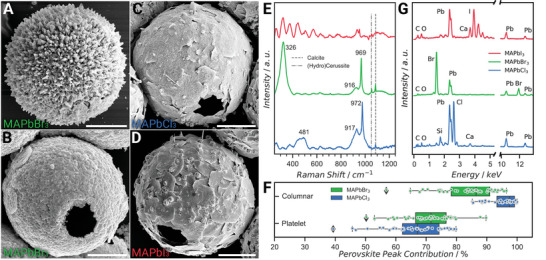
The second conversion step – the formation of methylammonium lead halide perovskites. The morphology of the shells after the second conversation step leading to the formation of A) column‐ and B) platelet‐covered methylammonium lead bromide (MAPbBr_3_) perovskite spheres. Focusing on the platelet‐covered morphology, similar structures are obtained by conversion into C) methylammonium lead chloride (MAPbCl_3_) and D) methylammonium lead iodide (MAPbI_3_). Conversion to lead halides was confirmed using E) Raman spectroscopy and G) Energy dispersive X‐ray analysis (EDX) exhibiting F) good conversion of an average of ≈70% perovskite peak area contribution for platelet structures and ≈90% for columnar structures in the case of MAPbBr_3_ and MAPbCl_3_. All scale bars are 5 µm.

In the second conversion step, the two obtained morphologies of cerussite and hydrocerussite were transformed into methylammonium lead halide perovskites. The anion exchange reaction was performed by exposing the lead carbonate nano‐structures to an excess of gaseous methylammonium halide (MAX) in a tube furnace at 120 °C and 70 mbar for 12 h. Whereas a good retention of the initial column‐like structure for cerussite was observed (**Figure** **3**A and Figure S2, Supporting Information), the platelet containing nano‐structure of hydrocerussite was lost and almost continuous perovskite spheres were obtained (Figure 3B–D). These morphological changes can be attributed to the fact that the dimensions of a structurally unstable reaction zone in anion‐ and cation exchanges depend on the size and charge density of the inwards diffusing ions.^[^ ^55^ ^]^ The dimensions of the reaction zone increase for smaller anions with higher charge density and thus, for example, morphological retention is more likely when working with iodide compared to chloride. Furthermore, as the hydrocerussite platelets exhibit sub 200 nm features, they are more likely to undergo morphological modifications, compared to the cerussite columns where the smallest dimensions are a few‐fold larger. Successful chemical transformation of these structures into all three variants of lead halide perovskites (MAPbX_3_ with X = Cl^−^, Br^−^, I^−^, MA = CH_3_NH_3_^+^) was again confirmed using spatially resolved Raman spectroscopy (Figure 3E). Prominent methyl rocking at 916–917 cm⁻^1^, as well as C‐N stretching signals at 971–972 cm⁻^1^ were detected for MAPbBr_3_ and MAPbCl_3_.^[^ ^56^, ^57^ ^]^ Additionally, methylammonium cage vibration at 326 cm⁻^1^ in the case of MAPbBr_3_ and MA torsion Raman signal at 481 cm⁻^1^ for MAPbCl_3_ were observed.

The conversion efficiency from lead carbonate to perovskite was determined from the ratio of integrated Raman signals of the internal (lead) carbonate vibration and the C‐N stretching modes for MAPbBr_3_ or MAPbCl_3_ demonstrating the high conversion of cerussite (≈90% perovskite peak contribution) and partial conversion of hydrocerussite (≈70% perovskite peak contribution) to perovskite (Figure 3F). Due to the semiconducting nature of the perovskite structure and the relatively narrow bandgap for MAPbI_3_ compared to the other perovskites, a significant fluorescent background impeded the detection of the weak Raman signal in this material. However, successful conversion was further supported using semi‐quantitative energy dispersive X‐ray spectroscopy (EDX), showing high contents of halides in all converted perovskites (Figure 3G).

**Figure 4:**
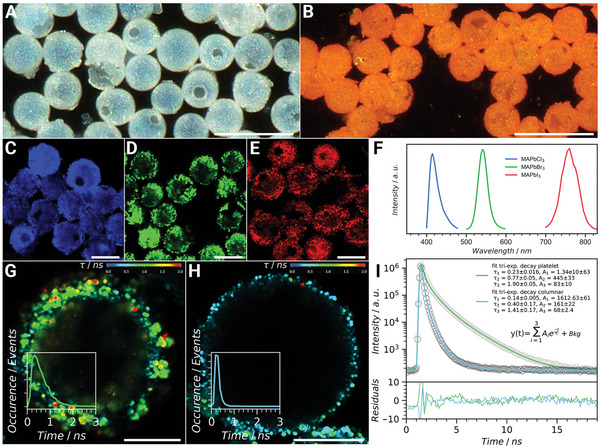
Electro‐optical properties of nanostructured perovskite shells. A,B) Optical images of *L. granifera* shells before and after conversion to MAPbBr_3_ perovskite, respectively, demonstrating the transformation from translucent calcite to a semiconducting material with electro‐optical properties that are responsible for the orange color of the transformed structures. C,D,E) Confocal fluorescence microscopy images from which F) emission intensity spectra show maxima at 415 nm (blue), 541 nm (green), and 760 nm (red) for methylammonium lead chloride, bromide, and iodide, respectively. G,H) Spatially resolved fluorescence lifetime images of platelet‐ and column‐covered shells, respectively, converted to MAPbBr_3_ with an inset showing the fluoresence decay curves indicative of the mean photon arrival times after the laser pulse. The images indicate the presence of small structures with long lifetimes (red) and an overall shift from longer (platelet‐covered shells) to shorter (columnar covered shells) lifetimes. I) The trend is confirmed by measuring the fluorescence decay of an ensemble of perovskite shells fitted with a tri‐exponential decay, indicating the presence of three distinct lifetime contributions. Scale bars: (A) 50 µm, (B) 50 µm, (C) 20 µm, (D) 20 µm, (E) 20 µm, (G) 5 µm, and (H) 5 µm.

The electro‐optical nature of the semiconducting MAPbX_3_ structures becomes apparent upon their interaction with light. Light microscopy images of the pristine calcitic cysts appear translucent due to the lack of electronic excitation upon interaction with the incident light (**Figure** **4**A). However, when converted, for example, to MAPbBr_3_, the mineral shells obtain an orange color as a result of the semiconducting nature of the material that absorbs light below a wavelength of 520 nm and emits photons at 536 nm (Figure 4B).^[^ ^58^ ^]^

An emission from all perovskite analogs imaged using a confocal laser scanning microscope emphasizes the complete and homogeneous transformation of the cysts to the corresponding perovskite materials. The chemically tunable emission peak wavelength is ranging from blue (X = Cl^−^), to green (X = Br^−^), to near‐infrared (X = I^−^) spectral regions (Figure 4C,D,E, respectively) corresponding to a change in the band gap energy (Figure 4F).

In contrast to static light emission, fluorescence lifetime measurements allow for quantification of the exciton couple lifetime, which is strongly dependent on the structure and size of the investigated perovskite materials.^[^ ^59^ ^]^ The spatially resolved fluorescence lifetime of platelet‐covered and columnar perovskite shells demonstrates the effect of nano‐structuring on their electro‐optical properties. In the case of MAPbBr_3_, platelet‐covered spheres show generally longer fluorescence lifetimes (Figure 4G), compared to their columnar analogs (Figure 4H), with lifetimes up to 2.5 ns (red points) corresponding to the edges of the flat crystals. The difference is also apparent in fluorescence decay fitted with a tri‐exponential decay function for both morphologies (Figure 4I). Comparing the lifetimes with the dominant amplitude we find that *τ*_1_ = 0.23 ns for platelet nano‐structures is almost two‐fold larger than the corresponding lifetime for columnar structures with *τ*_1_ = 0.14 ns. This emphasizes the capacity to tune the electro‐optical characteristics of the obtained materials by controlling their architecture at the nano‐scale.

## 3 Conclusions

In this work, we establish a scalable production route of nano‐architectured perovskite spheres with controllable morphologies and, by extension, their electro‐optical properties, using biogenic matter as the source material. Sustainable cultivation of unicellular calcitic algae with numerous other morphologies, crystallographic textures, and intricate ultrastructures on the nano‐ and the meso‐scales, such as coccolithophores and dinoflagellates, is well developed. Therefore, the presented approach can be used for the mass production of nano‐architectured perovskites with desired physical characteristics that are inherited from the vast pool of structural and textural properties provided by unicellular calcitic algae.

## 4 Experimental Section

### Cultivation of L. granifera

Cultures of *Leonella granifera* (strain 4751 B) were provided by the Culture Collection of Algae at the University of Cologne (CCAC). The artificial seawater (f/2) medium was prepared on the base of Tropic Marine Sea Salt at a concentration of 35 g L⁻^1^ instead of filtered seawater. Pre‐cultivation was performed in 50/250 mL filter‐cap cell culture flasks (Cellstar, Greiner bio‐one) with a sterile filter (0.22 µm, Corning) until a dense cell culture (10^5^ ‐10^6^ cells mL⁻^1^) was obtained. Growth was concluded either in 1 L f/2 in a Fernbach flask using 10 vol% cell culture or a 20 L polycarbonate carboy. A Flohr Instruments PLG 400 (28 °C) equipped with led lamps (14 h/10 h day/night cycle, light intensity: 50–100 µmol m⁻^2^ s⁻^1^) was used to provide constant growing conditions. Culture growth of a 20 L batch took ≈4–6 weeks.

### Algae Preparation

The algae were harvested by centrifugation using an Eppendorf Centrifuge 5810R at 3000 rcf and cleaned by redispersing the algae pellet in distilled water. This process was repeated three times. To remove organic material, the harvested algae were washed in a 5 vol% solution of sodium chlorite (NaClO_2_, 6–14%, Merck), water, and acetone thrice with intermediate centrifugation at 3000 rcf and dried from acetone using a desiccator. Residual organics were removed by heating the algae to 600 °C in an open atmosphere using a Carbo Lite T1 tube furnace. Using Raman spectroscopy, retention of the calcite phase was ensured and no calcium oxide was detected.

### Cation Exchange (First Conversion Step)

To convert the cleaned algae calcite into lead carbonate, the algae were placed in a 1.5 mL Eppendorf Tube. A stock solution of 24 m lead perchlorate (PbCl_2_O_8_·3H_2_O, 99%, Thermo Scientific) in ultrapure water (18.2 MΩcm) was mixed in a volume ratio of 10:1 and 3:5 with 1 m NaOH(aq) for the synthesis of cerussite (PbCO_3_) and hydrocerussite (Pb_3_(CO_3_)_2_(OH)_2_), respectively. 100 µL lead perchlorate solution was added to the algae and centrifuged at 2000 rcf for 17 min until maximum conversion. The reaction was stopped by adding 1 mL ultrapure water, centrifuging for 2 min at 2000 rcf, and consequently cleaning with 1 mL ultrapure water with consecutive centrifugation. The cleaning process was repeated three times. The converted algae were dried at 60 °C in an oven and could be stored in a dry environment.

### Anion Exchange (Second Conversion Step)

The lead carbonate structures were converted to MAPbX_3_ perovskite in the middle of a single‐zone tube furnace (GeroLite T1). The reactant methylammonium halide (MAX with X = Cl, Br, I) was placed in an alumina boat which was inserted into the quartz tube. The tube was freed of atmospheric air by reducing the pressure below 0.1 bar followed by flushing with argon. This process was repeated four times. For the anion conversion, the pressure was kept at 70 mbar while maintaining a 1 L min⁻^1^ argon flow. The temperature was increased to 120°C by first increasing the temperature to 80 °C at 5 K min⁻^1^ and consequently to the final temperature at 1 K min⁻^1^ steps. The reaction was stopped after 12 h by switching off the oven, and allowing the system to cool to room temperature. Perovskites were stored under a dry argon atmosphere.

### Scanning Electron Microscopy (SEM)

Imaging of the lead carbonate and perovskite structures was performed using a Scios Dual Beam FIB/SEM (FEI/Thermo Fisher) in high‐vacuum conditions with prior Pt/Pd‐coating.

### Electron Backscatter Diffraction (EBSD)

Electron Backscatter Diffraction (EBSD) data were collected using a Hikari Super EBSD (EDAX) system on a Scios Dual Beam FIB/SEM (FEI/Thermo Fisher). To minimize damage to the specimen surface by the electron beam, we used a low current of 1.6 nA and a voltage of 15 kV. EBSD patterns were processed using neighbor pattern averaging indexing. MTEX, a free crystallographic texture analysis software, was used for analysis.

### Energy‐Dispersive X‐Ray Spectroscopy (EDX)

Elemental composition was obtained with a Scios Dual Beam FIB/SEM (FEI/Thermo Fisher) scanning electron microscope equipped with an Octane Elite Super Silicon drift detector. An accelerating voltage of 30 kV and an electron current of 1.6 mA were used. To avoid charging, samples were Pt/Pd‐coated.

### Raman Spectroscopy

Spatially resolved Raman spectra were obtained using a Renishaw confocal Raman microscope equipped with a 532 nm (Nd:YAG, 50 mW, Renishaw) and a 785 nm (Diode Laser, 300 mW, Renishaw) laser and a 20x objective lens. Integration time was set to 0.5 s and 20 acquisitions averaged per algae. For lead carbonate structures, the 532 nm laser was used at 10% full laser power. In order to prevent electronic transitions leading to a strong fluorescent background in the perovskite samples, the 785 nm laser was used at 10% maximum laser power. Background subtraction was achieved using Asymmetric Least Squares Smoothing with a 10th degree polynomial. Raman peaks were fitted and numerically integrated using a Voigt profile using an in‐house Python script.

### Confocal Photoluminescence (PL) and Fluorescence Lifetime Imaging (FLIM)

Lifetime images were acquired and calculated using an inverted SP8 FALCON confocal laser‐scanning microscope (Leica Microsystems) using a 63x/1.2 NA water immersion objective with fluorescence excitation by a tunable supercontinuum white light laser pulsed at 40 MHz (NKT Photonics) using Leica Application Suite X (version 3.5.7.23225) and LAS X SMD FLIM (version 3.5.6). Fluorescence decays were fitted using a three‐exponential decay model convoluted with the instrumental resolution function and the instrumental background. Images of amplitude‐weighted mean lifetimes were exported.

Lambda detection scan series of images and corresponding intensity histograms of the chemically modified algae were acquired and exported using a confocal laser scanning microscope STELLARIS 8 (Leica Microsystems) with a tunable supercontinuum white light laser (NKT Photonics) and a 405 nm pulsed laser line (LD‐H‐D‐C405, PicoQuant) and a 16x/0.6 immersion adjustable objective and a 40x/1.2 water immersion objective using Leica Application Suite X (version 4.4.0.24861).

### Transmission Electron Microscopy (TEM)

TEM was conducted using a JEOL JEM F200 transmission electron microscope operated at 200 kV acceleration voltage and equipped with a Gatan OneView camera. Selected area electron diffraction patterns were collected using a selected area aperture of 50 µm diameter.

## Conflict of Interest

The authors declare no conflict of interest.

## Supporting information

Supporting Information

## Data Availability

The data that support the findings of this study are available from the corresponding author upon reasonable request.
