# Supplementary material for: Recruiting Unicellular Algae for the Mass Production of Nanostructured Perovskites
Source: Adv Sci (Weinh). 2023 Feb 12;10(11):2300355. doi: 10.1002/advs.202300355 (PMC10104627; doi:10.1002/advs.202300355)
Supplement: Supplementary file 1 — Supporting Information [file ADVS-10-2300355-s001.pdf]

SUPPORTING INFORMATION

Recruiting unicellular algae for the mass production of nanostructured perovskites

Lucas Kuhrts[1], Lukas Helmbrecht[2], Willem Noorduin[2,3], Darius Pohl[4], Xiaoxiao Sun[5], Alexander Palatnik[6], Cornelia Wetzker[7], Anne Jantschke[8], Michael Schlierf[1,9], Igor Zlotnikov[1]*

[1] B CUBE - Center for Molecular Bioengineering, Technische Universität Dresden, Germany

[2] AMOLF, Amsterdam, The Netherlands

[3] Van ‘t Hoff Institute for Molecular Sciences, University of Amsterdam, Amsterdam, The Netherlands

[4] Dresden Center for Nanoanalysis (DCN), Center for Advancing Electronics Dresden (cfaed), Technische Universität Dresden, Germany

[5] Helmholtz-Zentrum Dresden Rossendorf, Dresden, Germany

[6] Dresden Integrated Center for Applied Physics and Photonic Materials, Technische Universität Dresden, Germany

[7] Light microscopy facility of the Center for Molecular and Cellular Bioengineering (CMCB), Technische Universität Dresden, Germany

[8] Institute for Geosciences, Johannes-Gutenberg-Universität Mainz, Germany

[9] Physics of Life, DFG Cluster of Excellence, TU Dresden, 01062 Dresden, Germany

[*] Corresponding author: igor.zlotnikov@tu-dresden.de

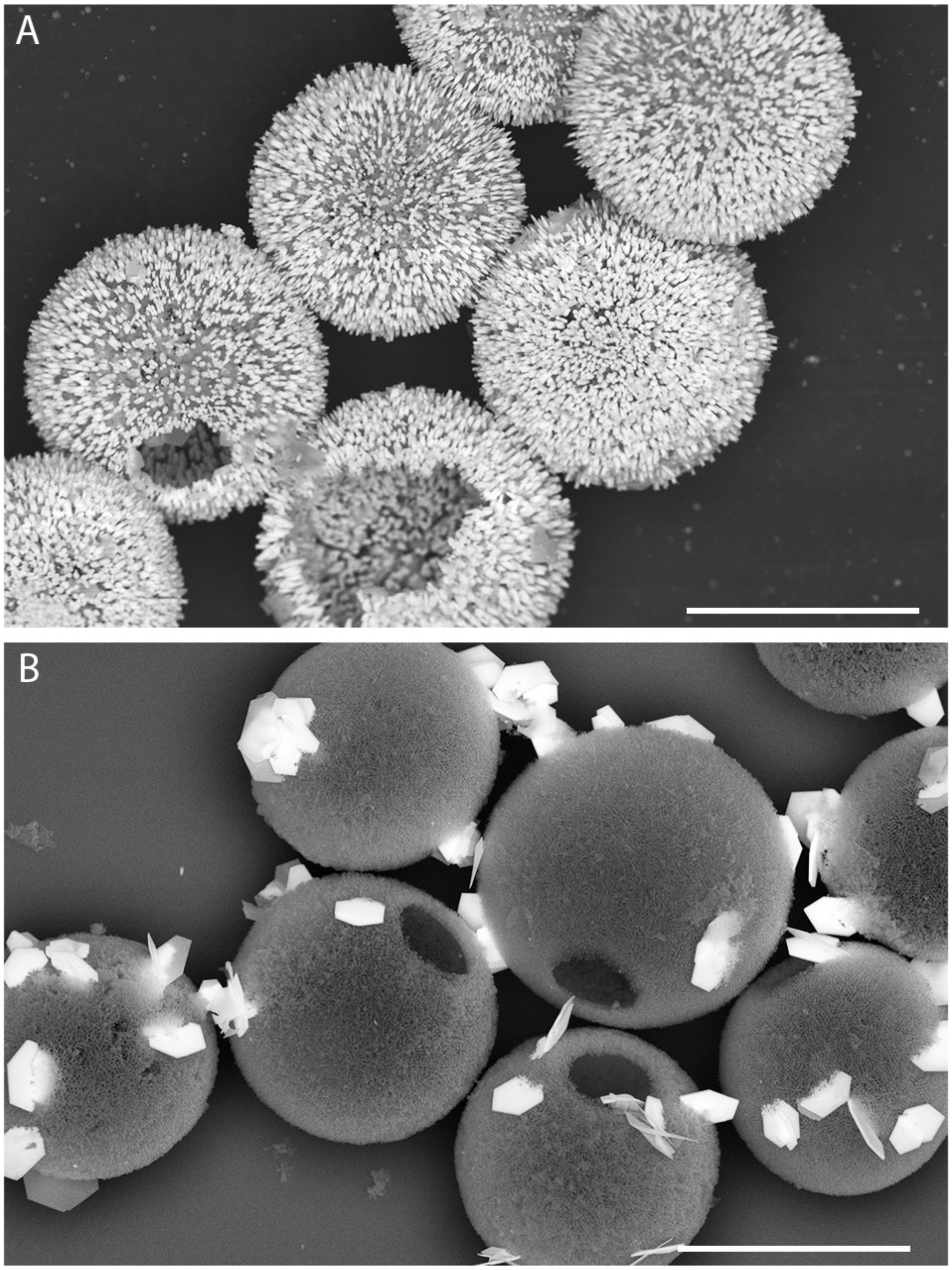

Figure S1: The first conversion step - the formation of lead carbonates. Backscattered electron microscopy images of (A) cerussite columns and (B) hydrocerussite platelets on top of the calcitic structure. Calcite appears as grey spheres on which bright lead carbonate particles are forming. All scale bars are 20 µm.

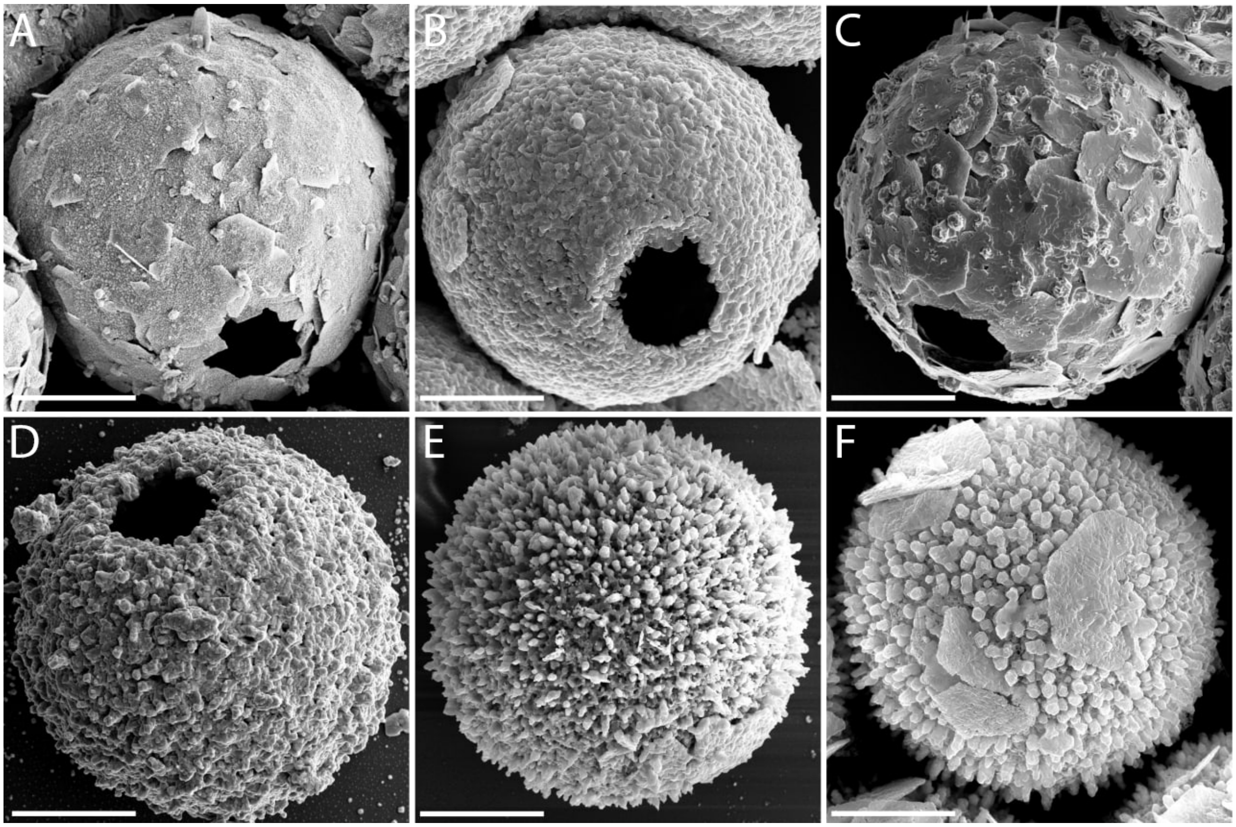

Figure S2: The second conversion step - the formation of methylammonium lead halide perovskites. The morphology of the shells after the second conversation step leading to the formation of (A-C) platelet-covered and (D-F) column-covered methylammonium lead chloride ($MAPbCl_3$), methylammonium lead bromide ($MAPbBr_3$) and methylammonium lead iodide ($MAPbI_3$) perovskite spheres, respectively. All scale bars are 5 μm.
